# Long-Term Skin Temperature Changes after Breast Cancer Radiotherapy

**DOI:** 10.3390/ijerph19116891

**Published:** 2022-06-04

**Authors:** Agnieszka Baic, Dominika Plaza, Barbara Lange, Łukasz Michalecki, Agata Stanek, Anna Kowalczyk, Krzysztof Ślosarek, Armand Cholewka

**Affiliations:** 1 Faculty of Science and Technology, University of Silesia, 75 Pułku Piechoty Street 1A, 41-500 Chorzów, Poland; armand.cholewka@gmail.com; 2 Radiotherapy Planning Department, Maria Skłodowska—Curie National Research Institute of Oncology Gliwice Branch, Wybrzeze Armii Krajowej Street 15, 44-102 Gliwice, Poland; dominikaplaza1@gmail.com (D.P.); krzysztof.slosarek@io.gliwice.pl (K.Ś.); 3 IIIrd Radiotherapy and Chemotherapy Department, Maria Skłodowska—Curie National Research Institute of Oncology Gliwice Branch, Wybrzeze Armii Krajowej Street 15, 44-102 Gliwice, Poland; barbara.lange@io.gliwice.pl; 4 Department of Radiation Oncology, University Clinical Center, Medical University of Silesia, Ceglana Street 35, 40-514 Katowice, Poland; lmichalecki@uck.katowice.pl; 5 Clinical Department of Internal Medicine, Angiology and Physical Medicine, Medical University of Silesia, Poniatowskiego Steet 15, 40-055 Katowice, Poland; astanek@tlen.pl; 6 Department of Physiotherapy, School of Health Sciences, Medical University of Silesia, Medyków Street 12, 40-752 Katowice, Poland; akowalczyk@sum.edu.pl

**Keywords:** radiation therapy, thermography, breast cancer

## Abstract

The aim of the study was to use thermal imaging to evaluate long-term chest temperature changes in patients who had previously been treated with radiotherapy. The examination with a thermal imaging camera involved 144 women—48 of them were patients after RT, 48 were females before breast cancer radiotherapy and the last group of participants were 48 healthy women. All patients (before and after radiotherapy) were divided into women after mastectomy and those after conservative surgery. In addition, the first group of women, those who had received radiotherapy, were divided into three other groups: up to 1 year after RT, over 1 year and up to 5 years after RT and over 5 years after RT. Due to this, it was possible to compare the results and analyse the differences between the temperature in the healthy and treated breasts. The comparison of obtained temperature results showed that the area treated by ionizing radiation is characterized by a higher temperature even a few years after the finished treatment. It is worth mentioning that despite the fact that the difference was visible on the thermograms, the patients had no observable skin lesion or change in color at the treatment site. For the results of the study provided for the group of healthy patients, there were no significant differences observed between the average temperatures in the breasts. The use of thermal imaging in the evaluation of skin temperature changes after radiotherapy showed that the average temperature in the treated breast area can change even a long time after treatment.

## 1. Introduction

Radiotherapy (RT) constitutes an essential part of the management of breast cancer in all stages of the disease [1,2,3,4,5,6]. The irradiated volume includes the breast with the primary tumor bed or the chest wall and the area of ipsilateral regional lymph nodes depending on the clinical stage of the disease and surgical treatment [7,8,9]. The patients are subjected to radical irradiation, which aims to destroy all tumor cells in the irradiated field and requires the use of a high total dose, administered in small daily doses (fractional doses), which allows for the repair and regeneration of healthy tissues. Conventional dosing for breast radiotherapy is 50 Gy in 2.0 Gy per fraction to the clinical target volume (CTV). The organs that are located in the vicinity of the irradiated volume, i.e., the lungs and the heart, are important in terms of the value of the deposited dose. These organs are called the critical organs or OARs (Organs at Risk) [10,11]. In general, radiation therapy in breast cancer is very well tolerated and patients can continue their normal life. Acute toxicity occurs within 3 months of radiation treatment and comprises fatigue, sore throat and radiation dermatitis. Long-term side effects of radiotherapy include an increased risk of heart or lung disease and secondary malignancy [12,13,14,15,16,17].

Nowadays, advance treatment planning techniques (IMRT—Intensity Modulated Radiation Therapy and VMAT—Volumetric Modulated Arc Therapy) allow for an essential reduction in these complications. However, the conformity of the dose distribution and protection of the surrounding tissues and organs stay the most important concerns regarding the treatment. One of the methods that may aid in the diagnosis of breast cancer is infrared thermography [18,19,20,21]. It has found wide application in many industrial fields, and is also successfully used in medicine. Thermal imaging cameras with specialized software enable the analysis of thermographic images of various diseases, in which the lesion is located on the surface of the skin or under its surface, which is both an advantage and a disadvantage of this diagnostic technique [22,23,24,25]. Thermographic imaging is based on the registration of “thermal” radiation emitted from the surface of the body as the effect of heat delivered to the surface of the skin as a result of internal tissue metabolism and vascular circulation. The main advantage of thermal imaging is the possibility of the functional evaluation of tissues, indirectly analyzing changes or the range of temperature changes in surface tissues [26,27,28,29]. Such a specific heat map can convey information about tissue metabolism, which is significantly accelerated in the case of many diseases, especially cancer [30,31,32,33,34]. Inspired by thermal imaging research, we decided to use its advantages, i.e., its safety and non-invasiveness, to assess the effects of radiotherapy treatment. The dose that the patient receives is not indifferent to him and often causes radiation reactions on the skin. This takes many forms, from slight reddening to a severe radiation reaction [35,36,37]. In current clinical practice it is only assessed visually. The application of thermal imaging may allow us to determine where it appears, how it changes over time and whether these differences are statistically significant. It may also allow us to assess how long it takes after the end of treatment for the body to return to equilibrium and whether this is possible at all.

The main aim of the study was to propose the use of thermal imaging to manage patients after radiotherapy for breast cancer. This publication sums up studies that have been conducted over the past few years.

## 2. Materials and Methods

During the control in clinics, 144 patients were examined including 48 women who had breast cancer radiotherapy, 48 women who had breast cancer surgery (conserving surgery or mastectomy) before radiotherapy and 48 healthy women. Patients received a total dose of 50 Gy at 2 Gy per day. Patients received adjuvant treatment, which is the use of radiation therapy in addition to surgery. The table (Table 1) below shows the three groups studied. The first two groups were further divided into patients after mastectomy and after conservative surgery.

This table (Table 1) includes the most important information about patient qualification: the mean age of this group, comorbidities, other cancers, local recurrence, oncology treatment and radiotherapy technique. First group of patients (after RT) were divided into 3 groups: up to 1 year after RT, over 1 year up to 5 years after RT and over 5 years after RT. Every subgroup included 8 patients. Then, the differences between the temperature in the healthy and treated breasts were analyzed. The study design was approved by the Bioethics Committee of the Oncology Center on 6 October 2016, which was confirmed by the opinion No. 38/2016.

All patients were examined in the same room, previously prepared for thermal imaging studies. It was characterized by a constant temperature of 22 ± 1 °C and air humidity from 40% to 45%. A FLIR System E60 thermal imaging camera was used with a detector resolution of 320 × 240 pixels, the thermal sensitivity of which is 0.05 K [21,22]. Each patient gave her written consent to participate in the study. Patients had to take off clothes and the bra and not perform any physical activity for a specified period of time (minimum 20 min) before the examination. There were no nursing or pregnant women in the study group. It should be emphasized that only a well-prepared patient, room and trained staff are allowed to obtain the reliable results and, more importantly, to analyze those results in detailed and accurate way [23,24,25]. The aim of the study was to assess the temperature parameters of the breast area in patients undergoing radiotherapy at various long-term intervals after treatment.

After the examination, the obtained thermograms were analyzed with the ThermaCam Researcher Pro 2.10 software (FLIR Systems AB, Danderyd, Sweden). Student’s t-statistical tests were performed (the confidence interval was 0.95) using the STATISTICA 10 (StatSoft, Kraków, Poland).

The area of healthy breast and treated breast was defined for each patient. Depending on the size and structure of the breasts, it was adapted to the anatomy of each patient.

## 3. Results

**Figure 1:**
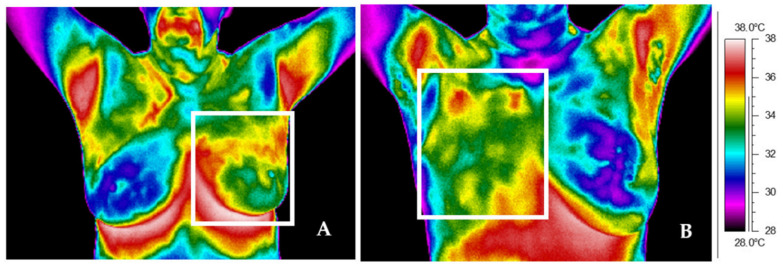
Thermal image of representative patient after surgery (**A**) and mastectomy (**B**) taken up to 1 year after the end of radiotherapy treatment.

**Figure 2:**
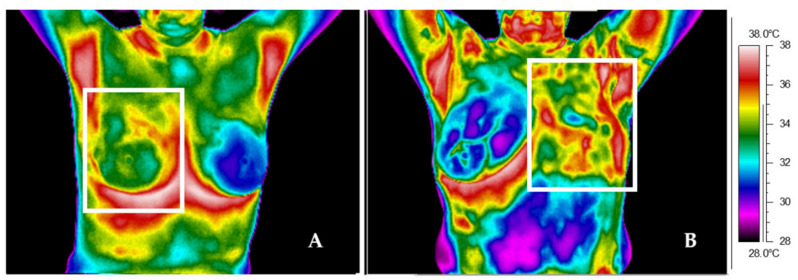
Thermal image of representative patient after surgery (**A**) and mastectomy (**B**) taken over 1 year and up to 5 years after the end of radiotherapy treatment.

**Figure 3:**
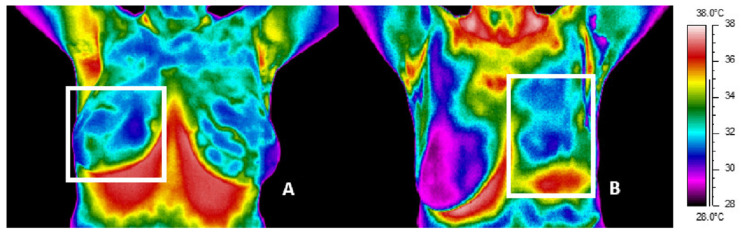
Thermal image of representative patient after surgery (**A**) and mastectomy (**B**) taken over 5 years after the end of radiotherapy treatment.

The paper compared thermograms of patients after surgery and mastectomy up to 5 years after radiotherapy. Thermal images for example patients after surgery (A) and mastectomy (B) are presented in Figure 1, Figure 2 and Figure 3. White frames determine the treatment area.

The thermograms (Figure 1A,B) were performed up to 1 year after the end of treatment. Figure 1A shows a thermal image for a patient after left breast surgery. The thermal asymmetry between the treatment and healthy breasts can be easily seen. The difference between the mean temperatures of the whole breast area is 1.0 °C. Figure 1B shows a thermal image for a patient after right breast mastectomy. The difference in mean temperature between the analyzed chest sides is 1.1°C.

Figure 2A,B shows thermal images performed over 1 year and up to 5 years after RT. Figure 2A presents a thermogram of a patient after right breast surgery where the difference between the breast temperatures is 1.3 °C, and Figure 2B shows a thermogram for a patient after left breast mastectomy and the mean temperature difference in the corresponding areas is 1.7 °C. The thermal asymmetry between the breasts in the temperature distribution is clearly visible. The treated area has a higher temperature than the healthy area.

**Figure 4:**
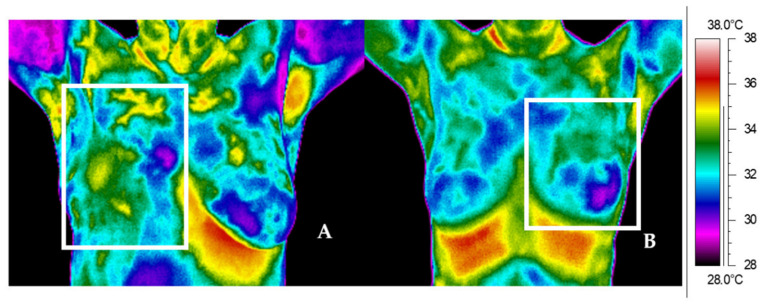
Thermal images for representative patient after mastectomy (**A**) and conserving surgery (**B**).

The last presented Figure 3A,B includes thermal images for patients that were taken more than 5 years after RT. Figure 3A shows a thermogram for a patient after right breast surgery for whom the mean temperature value in the left breast was 33.3 °C; however, the mean value in the treated right breast was higher by only 0.1 °C and was 33.4°C. On the other hand, Figure 3B shows a thermogram for a patient after left breast mastectomy for whom the temperature difference between the studied sides of the chest is 0.4 °C. Below (Figure 4) are sample thermograms for a patient after mastectomy (A) and for a patient after conserving surgery (B) before RT.

**Figure 5:**
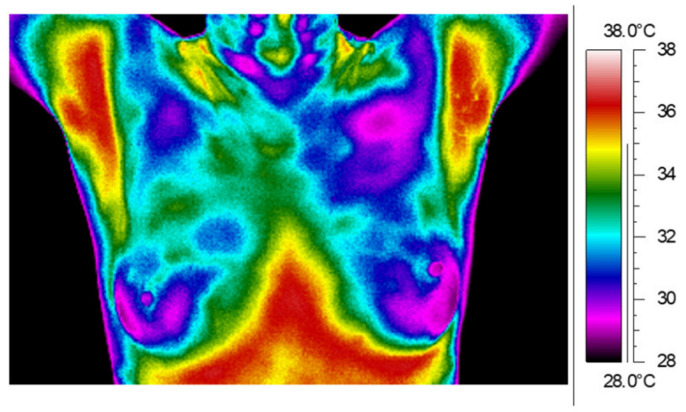
Thermal image for representative healthy woman.

The thermogram does not show any disproportions; it resembles a thermogram of a healthy patient (Figure 5).

The research group after RT was divided into three subgroups defined by the time that has passed after completed radiotherapy. In the table below (Table 2) the division into groups and the temperature difference between the irradiated area and a healthy breast are collected. The temperature differences between the breasts of healthy women, women after conserving surgery before RT and women after mastectomy before RT are also presented below.

**Figure 6:**
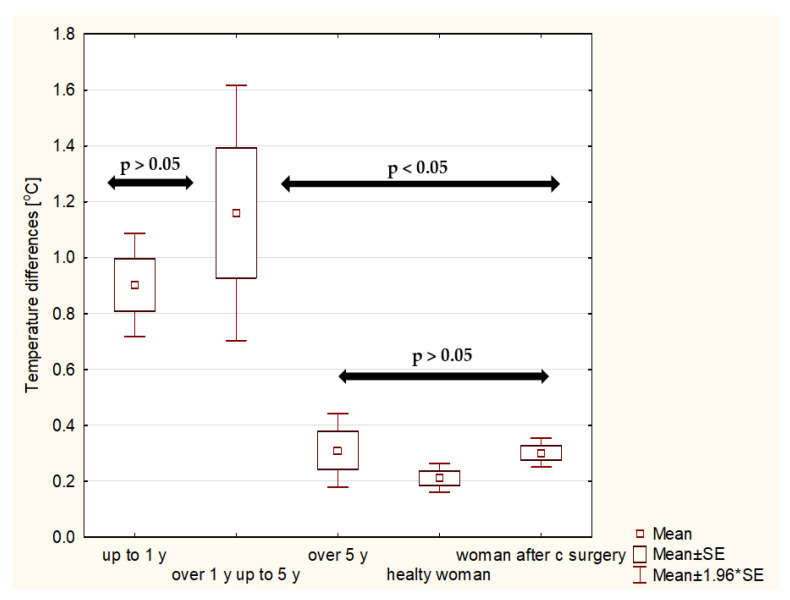
The average temperature difference for irradiated patients after surgery obtained between breasts in three time intervals: up to 1 year ago, over 1 year and up to 5 years and over 5 years ago for patients after surgery. The results for healthy women and women after conserving surgery have been added to the comparison.

**Figure 7:**
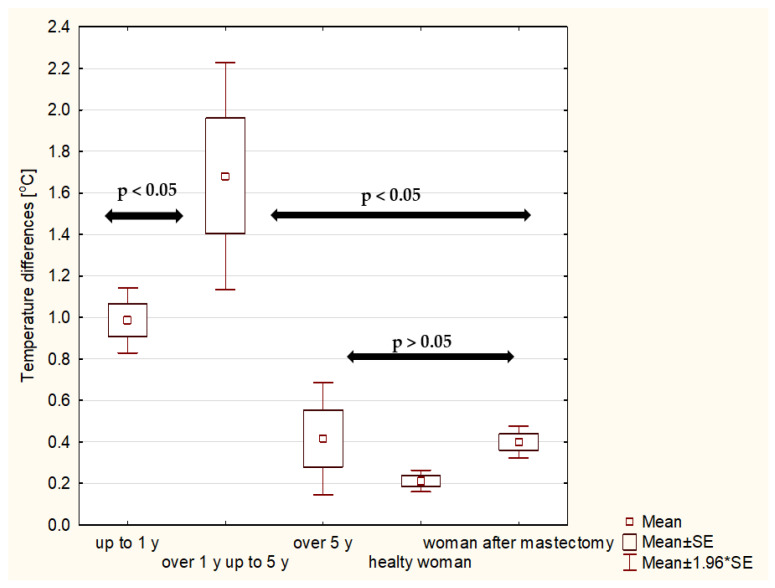
The average temperature difference for irradiated patients after mastectomy obtained between breasts in three time intervals: up to 1 year ago, over 1 year and up to 5 years and over 5 years ago for patients after mastectomy. The results for healthy women and women after mastectomy have been added to the comparison.

It should be underlined that the highest temperature difference was observed in the patients who were irradiated over 1 year and up to 5 years after RT. The temperature differences between the groups up to 1 year after radiotherapy and over 1 year and up to 5 years after radiotherapy are not statistically significant, while in comparison with the group over 5 years after radiotherapy, statistically significant differences were obtained (*p* < 0.05) (Figure 6 and Figure 7). Another analysis carried out in the study was the comparison of temperature differences in the skin of irradiated patients with a control group of healthy women, women after conserving surgery and women after mastectomy. Statistical significance (*p* < 0.05) was confirmed as shown in Figure 6 and Figure 7, respectively. The arrows in the graph indicate statistical significance between the study groups.

**Figure 8:**
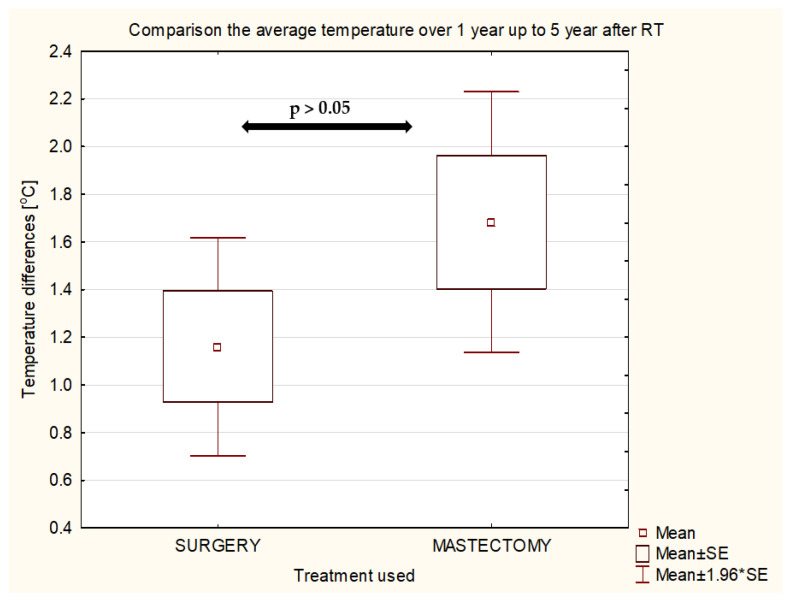
Comparison of mastectomy and surgery for the group of patients over 1 year and up to 5 years after radiotherapy.

It can be observed that the average temperature differences between the breasts in the group of patients irradiated over 1 year and up to 5 years after treatment were about 1.20 °C for patients after surgery and 1.68 °C for those after mastectomy. On the other hand, those differences decreased for patients over 5 years after RT to 0.30 °C and 0.42 °C, respectively. In the group of healthy patients, no disproportions between the average temperatures in the breasts were observed. The mean value of this difference was about 0.21 °C. The temperature difference was 0.30 °C for patients after conserving surgery and 0.40 °C for patients after mastectomy. It can be observed that the values in each time interval after radiotherapy are slightly higher for patients after mastectomy (Figure 8), but the values are not statistically significant.

## 4. Discussion

Late skin lesions appear within a few months or years after the end of treatment with ionizing radiation. It usually takes the form of: skin hypo- or hyperpigmentation, hyperkeratosis, dryness, telangiectasia, skin atrophy and discontinuity [38,39,40]. Skin changes after radiotherapy for breast cancers of varying severity are found in practically 90% of women. It is not a serious complication, but sometimes its recovery takes a long time and is onerous for the patient. The main principle in the treatment of skin complications is proper hygiene and care for the irradiated area.

The severity of acute and late skin side effects of breast cancer irradiation is dependent on many physical and clinical factors, especially the total radiation dose, fractionation, irradiated volume, patient’s age, comorbidities and genetic predisposition. In the course of radiotherapy, skin toxicity remains an important clinical problem for many patients [41,42,43,44,45,46,47].

However, it is difficult to estimate the frequency and intensity of these adverse effects.

Various classification systems are available. The most frequently used are the RTOG (Radiation Therapy Oncology Group)/EORTC (European Organization for Research and Treatment of Cancer) scale, the LENT (Late Effects Normal Tissue Task Force) SOMA (Subjective, Objective, Management, Analytic) scale and the Common Toxicity Criteria scale, the Dische scale, the Franco–Italian Glossary scale and the NCI (National Cancer Institute) system [3]. Radiation-induced tissue injury occurs on a functional, cellular and gross level. RT induces the generation of short-lived free radicals, irreversible breaks in cellular DNA, and an inflammatory response caused by a proinflammatory cytokine cascade (IL-1, IL-3, IL-5, IL-6, TNF-a), chemokines (IL-8, eotaxin, CCR receptor), receptor tyrosine kinase and adhesions molecules (ICAM-1, VCAM, E-selectin). Tissue healing is impaired by the destruction of the basal keratinocytes and each additional exposure to RT results in further direct tissue injury, inflammation and impaired epithelial regeneration, all of which leads to the development of acute radiation injury [16,38]. Such reactions lead to metabolism changes in the treated tissue not only on the surface but also beneath the skin. Thus, it results in body temperature changes that have been observed in conducted studies and shown in results even a few years after radiotherapy. Rarely, acute radiation fails to heal and, consequentially, late changes from RT may develop, which include chronic wounds and skin necrosis [16]. Differently, chronic radiation dermatitis is a true late-stage reaction that develops months to years after exposure to RT. The condition may develop in patients who only experienced minimal acute radiation dermatitis and so may develop in near-normal-appearing skin [5]. Chronic radiation dermatitis may explain our observation of the differences in skin temperature in patients over 5 years after breast radiotherapy. However, it is difficult to define the temperature rise pathogenesis. The development of chronic radiation dermatitis is related to the cytokine TGF-β, which is a regulatory protein controlling the proliferation and differentiation of many cell types, wound healing and the synthesis of extracellular matrix proteins in the normal tissue inflammatory response and development of late radiation-induced fibrotic changes [6]. The pathogenesis of telangiectasia development is thought to be in part due to acutely damaged microvasculature and the production of platelet-derived growth factor (PDGF) and fibroblast growth factor by damaged cells [16]. The development of radiation-induced fibrosis is induced by inflammation following RT and continues for months up to years [5]. TNF-α, IL-6 and IL-1 are related to the inflammatory response, while TGF-β and PDGF stimulate fibroblast activity and enhance the production of extracellular matrix proteins [17,39]. These changes and radiation-induced alterations of the microvasculature provide significant late toxicity of RT [5]. Moreover, it influences the metabolism in tissue so its range as well as magnitude may be easily detected in thermal imaging. However, it is not specifically used for just one form of the mentioned processes but will indirectly describe most or even all of them. One way or another thermal imaging may be helpful in the estimation of the range of occurrence of processes that lead to temperature change.

In should be underlined that the obtained results confirmed that temperature changes in patients after radiotherapy are variable and dependent on time. Moreover, it can be concluded that it is an appropriate solution to divide patients into different groups defined by periods of time that have passed since their treatment. In the study group, the difference between the healthy and irradiated breast was the greatest in the studied period over 1 year and up to 5 years after the end of treatment. After this period, the temperature differences decreased. It is worth noting that in patients who were treated with radiotherapy more than 5 years ago, the difference between the treated and healthy breasts for patients after surgery (0.30 °C) and after mastectomy (0.42 °C) were similar to the differences in healthy patients (0.21 °C). These results are consistent with the information sourced from the available literature and confirm an increase in temperature in the treated breast area with the appearance of late skin lesions from several months to several years after the end of treatment. The differences in the mean temperatures for patients after mastectomy were higher than the values for patients after surgery in each time interval. This is probably related to the differences in target volumes in postmastectomy patients (CTV includes skin). In addition, the cause could be due to the utilization of the bolus, i.e., a tissue-equivalent material placed on the postmastectomy scar in order to increase the skin dose, which may lead to a higher risk of radiation dermatitis.

Special patient care and observation with the use of a thermal imaging camera makes it more possible to define precisely the duration of a patient’s recovery and to specify the exact time at which imaging the changes resulting from the action of ionizing radiation on the treated tissue is possible.

## 5. Conclusions

The use of thermal imaging in the evaluation of skin temperature changes after radiotherapy showed that the average temperature in breast area varies for a long time after breast cancer radiotherapy. The biggest temperature difference was observed in the period over 1 year and up to 5 years after radiotherapy. However, more than five years after the end of treatment, the differences between the thermal asymmetry (mean temperatures) between the breasts were at their lowest level. Additionally, it seems that the temperature stabilizes and is close to values obtained in the not-irradiated body side, although the values were still higher than in the examined healthy women. It is necessary to expand the research group in order to confirm the effectiveness of the use of thermovision in the control of patients after radiotherapy.

## Figures and Tables

**Table 1:** Summary of patient eligibility information for the research.

Groups	Patients	Amount	Age	Comorbidities	Treatment	Other Cancers	Local Recurrence	Radiotherapy Technique
Patients after RT	After mastectomy	24	56 ± 9 years	no	adjuvant	no	no	Dynamic -IMRT/VMAT
	After surgery	24	54 ± 5 years	no	adjuvant	no	no	Dynamic -IMRT/VMAT
Patients before RT	After mastectomy	24	52 ± 6 years	no	adjuvant	no	no	-
	After surgery	24	59 ± 8 years	no	adjuvant	no	no	-
Healthy	Healthy women	48	50 ± 7 years	no	-	-	-	-

**Table 2:** The average temperature difference between the irradiated area and a healthy breast depending on the irradiation time.

Patients after Surgery		Patients after Mastectomy	
Time after radiotherapy	Temperature difference between irradiated and healthy breast (°C)	Time after radiotherapy	Temperature difference between irradiated and healthy breast (°C)
Up to 1 year	0.90 ± 0.21	Up to 1 year	0.99 ± 0.14
Over 1 year up to 5 years	1.20 ± 0.25	Over 1 year up to 5 years	1.68 ± 0.27
Over 5 years	0.30 ± 0.11	Over 5 years	0.42 ± 0.13
Temperature difference between breasts for healthy women (°C)	Temperature difference between breasts for women after conserving surgery (°C)		Temperature difference between breasts for women after mastectomy (°C)
0.21 ± 0.05	0.30 ± 0.09		0.40 ± 0.12

## Data Availability

Not applicable.
